# The Profile of Saudi Nursing Workforce: A Cross-Sectional Study

**DOI:** 10.1155/2017/1710686

**Published:** 2017-10-29

**Authors:** Mohammad Alboliteeh, Judy Magarey, Richard Wiechula

**Affiliations:** ^1^University of Hail, Hail, Saudi Arabia; ^2^University of Adelaide, Adelaide, SA, Australia

## Abstract

**Introduction:**

The Royal Monarchy in Saudi Arabia decreed that all sectors of the workforce would be subject to a policy of “Saudisation” to reduce the reliance on the expatriate workforce and to reduce the unemployment rate of Saudi nationals (Al-Mahmoud et al., 2012).

**Methodology:**

A cross-sectional design was chosen to investigate the research questions. The population of this study comprised Saudi Registered Nurses working in MOH hospitals in Riyadh which is the main health care provider in Saudi Arabia (Aboul-Enein, 2002; MOH, 2009).

**Results and Findings:**

A total number of 1,198 questionnaires were distributed and 61.2% (*n* = 741) were returned. The findings of the study showed that the questionnaires were collected from an equal portion of the study locale and that a sample of 741 is enough to create a strong conclusion and answer the problem set in this study and all the questions in the study have been provided with answers with enough data and literatures to supports its findings.

**Conclusion and Recommendations:**

The results indicate that an increase in the recruitment of Saudi males may simply reflect cultural issues such as gender specific facilities and the Saudisation program's nondiscriminatory approach to employment of both genders into nursing.

## 1. Introduction

In 1992 the Royal Monarchy in Saudi Arabia decreed that all sectors of the workforce would be subject to a policy of “Saudisation.” This policy aimed to reduce the reliance on the expatriate workforce and to reduce the unemployment rate of Saudi nationals by restricting employment of foreign workers, reserving some jobs for Saudi citizens only and creating new jobs for Saudis [1]. Concurrently there has been an increase in the number of nursing schools and government hospitals being established [2]. The policy has resulted in a rapid increase in the number of Saudis entering the nursing workforce. In just one decade the percentage of Saudis in the nursing workforce increased from 22.3% to 50% [2]. However, unlike other developed countries, men comprise 25% of this workforce and 50% of all Saudi nurses [2]. This is a unique and relatively new phenomenon; previously there have been few Saudi males in the nursing workforce.

In the study of Angelo Dante et al. (2014), the authors mentioned that creating awareness and providing knowledge about a particular profession, like nursing, for instance, should start as early as when an individual person can already understand and can make decisions about his career in the future. Moreover, an accurate and precise information about a particular profession should be thoroughly provided and instilled to them thus to allow the individual to form judgment and proper decision on his or her career options based on a personal motivation, aspiration, and talents.

The literature now indicates that a major issue is the retention of these new nurses. Studies have shown that many nurses express dissatisfaction about their workplace and that decision makers are unable to meet their needs ([3, 4], Almalki 2011). In addition the nursing profession continues to suffer from a poor image in Saudi society [4]; this may also impact on retention of nurses in the workforce.

Because the gender profile is in direct contrast to that in other developed nations and there is a dearth of literature investigating this issue, the primary purpose of this study was to understand the factors that have led to an influx of male nurses into the sector, by comparing the views of both male and female nurses currently working in the field. In addition there is little literature published on factors impacting on retention. Thus the research questions were “What factors motivate Saudis enter the nursing workforce and are these different for males and females?” and “What are Saudi Nurses' perceptions of the profession and their future plans?”

## 2. Materials and Methods

A cross-sectional design using a questionnaire was chosen to investigate the research questions. The collection of data took place in hospitals of the Ministry of Health (MOH) in Riyadh city, Saudi Arabia, commencing December 12, 2013, and culminating on April 2014. Until the authors decided to have the study published in a peer reviewed journal, the search for related literatures and studies that support its findings continued. During the time of data collection, three general hospitals and two medical cities (a group of specialist hospitals) in Riyadh city were managed by the Ministry of Health. Each of these medical cities comprised different specialised hospitals. The population of this study was all Saudi Registered Nurses; the sample comprised Saudi Registered Nurses working in MOH hospitals in Riyadh. The MOH is the main health care provider in Saudi Arabia, providing 60% of health care to both Saudis and non-Saudis and its health facilities and activities are concentrated in Riyadh [5, 6].

The study was approved by the Human Research Ethics Committee at the University of Adelaide and the Department of Medical Research at the Ministry of Health in Saudi Arabia. In addition, ethical approval was sought from the management of each of the individual hospitals included in the study. Return of the completed questionnaire was considered to constitute consent and the anonymity of the participants was maintained with no individually identifying data collected.

Information regarding the study was presented to all nursing managers (NM) and head nurses (HN) in each hospital. The questionnaires were distributed to nursing department representatives, who then gave them to all Saudi nurses in their units via communication boxes. To draw attention to the study, a colourful flyer in Arabic was posted on the staff notice board of each unit and where possible short presentations were given to nursing staff during the morning staff unit meetings. Labelled collection boxes were distributed to all units for confidential collection of the questionnaire.

### 2.1. Tool Development

The questionnaire was developed and constructed in English and then translated into Arabic (see Appendix  2 in Supplementary Material available online at https://doi.org/10.1155/2017/1710686). A panel comprising an expert in gender and nursing workforce studies, two nursing academicians, and several experts in questionnaire design were consulted to consider face and content validity of the tool before it was translated into Arabic. It was then checked and back translated by a linguistic expert and two Saudi academics to further ensure face and content validity.

The tool consisted of four sections, Section A of the questionnaire contained ten items related to demographic and occupational information, and Section B comprised 17 items and was based on the literature identifying the most common motives for students and registered nurses to enter the profession [7–12]. Section C contained 10 items concerning perceptions of nursing measured using a 5-point Likert scale. This section was based on the results of published studies which investigated this issue [10, 13–16]. Finally, Section D was designed to measure the future plans of Saudi nurses in terms of intention to leave the profession. For the participants to answer accurately the questionnaire they were asked to choose from a 5-rating scale where 1 would mean extremely disagree, 2 disagree, 3 cannot decide, 4 agree, and 5 extremely agree.

The questionnaire was piloted by distribution to 15 Saudi nurses for testing in the target setting; however, no changes were required. The responses from the nurses who piloted the questionnaire were not included in the final study. Descriptive statistics were used to analyse the demographic information. Chi square was used to explore the relationships between categorical variables, primarily gender and other variables. IBM SPSS (Version 18) was used for analysis. It has been mentioned in the study that the tool was developed by the researcher. While it is true that creating or developing a questionnaire should undergo the same rigor as other tools that have been widely recognized and internationally by most researchers, the tool used in this went through the validity and test of rigor like other tools used in scientific studies. The preceding processes thoroughly described the processes the tool went through before and was even pilot tested to assure its validity, accuracy, and reliability.

## 3. Results

A total number of 1,198 questionnaires were distributed and 61.2% (*n* = 741) were returned. Though it shows that only 61.2% of the questionnaires were returned back, this does not affect the conclusiveness of the findings of this study for some reasons. First among these is that the questionnaires were collected from an equal portion of the study locale which therefore makes the impression that all the participants regardless of their affiliation or workplace were represented in the study. Second, a sample of 741 is enough to create a strong conclusion and answer the problem set in this study. Third, all the questions in the study have been provided answers with enough data and literatures to supports its findings. Most respondents were female (*n* = 554, 74.8%) with 25.2% (*n* = 187) being male. The mean age of the respondents was 27.1 years (SD, 4.84) (range 20–48 years). There was no statistically significant difference between males and females in regard to age. There was an almost equal distribution among the respondents in regard to marital status, with 49.5% (*n* = 366) being married. The majority of respondents in this study, 64.4% (*n* = 477), had no children, whereas 25.2% (*n* = 187) had one or two children and only 10.4% (*n* = 77) had more than two children. More than three-quarters of the respondents were from the central region, 76.6% (*n* = 567), where Riyadh city is located. There were no statistically significant differences between the genders for any demographic factors.

Respondents were asked to indicate their highest qualification in nursing. As can be seen from Table 1 most held a diploma; however, there was a statistically significant difference (*p* = 0.001) between males and female respondents, with females holding lesser qualifications than males. As can be seen from Table 2, the majority of respondents were relatively inexperienced with nearly three-quarters having less than 5 years' experience. The majority of respondents (86.8%, *n* = 644) held a clinical position. Half of the remainder (6.6%, *n* = 49) held an educational position and the other half (6.6%, *n* = 49) were in management positions. There was no difference between genders in relation to positions held. The greatest proportion of respondents, 82% (*n* = 600), were practicing nursing in medical, surgical, emergency, midwifery, and out-patient departments. The rest are practicing in other areas such as ICU, mental health, and paediatric care. There was no statistically significant difference between males and females in regard to area of practice.

### 3.1. Motivation to Enter the Nursing Profession

There was a strong level of agreement for items (1)−(11) (Table 3) with 63.4–94.4% of participants indicating they either agreed or strongly agreed with the statements. The items with the strongest agreement were mostly altruistic such as wanting to help people, wanting to work in a caring profession, and wanting to help others cope with illness. Items regarding job security, flexibility, and career advancement had somewhat less agreement although still rated strongly. The influence of others was not a strong motivator. Half of the participants agreed or strongly agreed that advice from family was a motivator although expectations of the family rated slightly less. Just over half indicated that a childhood desire was a motivating factor. There was a statistically significant difference between the genders for the reasons “it was a childhood desire” (*p* = 0.00), male 45% and females 60.7% (*n* = 332), and “it was a family expectation”; for male respondents the rate of agreement, 33% (*n* = 61), was lower than for female respondents, 48% (*n* = 262) (*p* = 0.01). There was also statistically significant difference, in relation to advice from friends and nurses (*p* = 0.01) with male respondents more motivated to become nurses after gaining advice from friends and nurses than females. There was also statistically significant difference in personal experience as a motive for respondents (*p* = 0.01) but overall the motivation was not strong for either gender.

### 3.2. Perceptions on Nursing

There was a strong level of agreement for items (1), (3), (6), (7), (9), and (10) (nursing is caring profession, requires physical activity, is a stressful career, offers variety, is well paid, and requires you to be away from home for a long time) with the majority of participants either strongly agreeing or agreeing with the statements. But for items (2) and (5) (nursing is for women and nursing does not require high academic qualifications) more participants disagreed or strongly disagreed. For items (4) and (8) (nursing is a profession that is subservient to doctors and is a respected profession) opinions were more equivocal. There were 4 items in which there was a statistically significant difference between genders. For the statement the profession is subservient to doctors, 47% (*n* = 88) of male respondents agreed or strongly agreed and only 40% of the females, (*n* = 200) agreed or strongly agreed with this statement (*p* = 0.00). For the statement that nursing does not require high academic qualifications 38% (*n* = 70) of males agreed or strongly agreed and 28% (*n* = 154) of females agreed (*p* = 0.002). For item (8) (nursing is a respected profession) 59% of males (*n* = 111) and 42% (*n* = 233) of females agreed or strongly agreed (*p* = 0.001). Most respondents (male 85.6%  *n* = 160, female 90.2%  *n* = 496) perceived nursing as a career that keeps them away from home for long periods of time. It was found that there is a statistically significant difference (*p* = 0.01) between male and female respondents in regard to this statement. The above description illustrates the perceptions of participants according to the their gender; Table 4 shows the perceptions of all participants.

### 3.3. Future Plans

The final section of the questionnaire was designed to investigate the nurses' future plans (Table 5). When respondents were asked whether they would prefer to work part time, almost half of the respondents, 49% (*n* = 356), agreed. Fifty-seven percent of these (*n* = 210) would like to work four days a week, 28% (*n* = 102) would like to work three days a week, and 6% would like to work one to two days per week. There were no statistically significant differences between genders. When respondents were asked whether they would like to work shorter shifts, 71.6% (*n* = 525) answered yes and 28% responded no. There was no difference between genders in regard to these items.

Nearly a quarter of the respondents, 23% (*n* = 167), indicated they intended to leave nursing within two years. There were no significant differences between the responses according to gender. Thirteen Likert items were designed to investigate the reasons respondents were planning to leave the profession in the next two years or would like to leave. For items (1), (2), (7), and (10) (my gender, dealing with the opposite sex, I am moving away, and I found a better job) the responses were equivocal. The majority of respondents disagreed with statements (3)–(7) and (13) (I feel other nurses are not comfortable with me, I feel uncomfortable dealing with the opposite sex, I feel uncomfortable dealing with patients of the opposite sex, I feel uncomfortable dealing with physicians of the opposite sex, and I am having difficulties in communicating in English), while for items (9), (10), and (12) (I will become a full time student, lack of promotion opportunities, and I have to work long hours) the majority of respondents agreed or strongly agreed with the statements. However, it is important to note that while for the majority of respondents indicated the gender issues are not important in influencing their decision to stay in nursing there are still a proportion of nurses for whom these are critical issues. Over a third of nurses agreed or strongly agreed with items (1), (2), (4), (5), and (6) (the reason I would leave is my gender, dealing with the opposite sex, dealing with nurses of the opposite sex, dealing with patients of the opposite sex, and dealing with physicians from the opposite sex).

There was only one item where there was statistically significant difference between the responses of males and females. This was for those who have the intention or would like to leave nursing, dealing with the opposite sex in the work place. The rate of agreement for males was 60% (*n* = 37) and for females 44% (*n* = 91) (*p* = 0.01). At the end of the survey the respondents to this study were asked to recommend changes to nursing which would alter their decision to leave the profession. Out of 741 participants, only a small proportion, 10% (*n* = 73), of the respondents answered this question. A content analysis (Table 6) was performed and the following themes were identified and ranked in order of frequency.

In the study “Nursing Student Plans for the Future after Graduation: A Multicentre Study” published in the International Nursing Review by A. Palese et al. (2016), it was found that nurses after graduation have different plans with regard to their career path. Some of the participants want to have a job right after nursing school; a portion of them want to emigrate to other countries and work while the rest aimed at pursuing further education.

## 4. Discussion

The study was designed to investigate the factors motivating Saudis to join the nursing profession and to determine if these were influenced by gender. The study also aimed to examine nurses' future plans to stay in the workforce and their perceptions of nursing as a profession. The results indicate that Saudi nurses are relatively young (mean age 27 years), inexperienced (77% working in the field for fewer than five years), and not highly educated (with 83% holding a certificate or diploma as their highest qualification). This is in contrast to nurses in Australia and most Western countries with the average age of nurses in Australia of 44.5 years [17], in Singapore 35 years, in Canada, the UK, and New Zealand 44 years, and in the United States 47 years [18, 19]. Regarding qualifications, in Australia 82% of registered nurses hold a Bachelor degree [17]. However, it should be noted that respondents to this study were exclusively registered nurses of Saudi nationality. These nurses have a very different profile from the expatriate nurses working within the Saudi system. The youth and inexperience of nurses in this study may be attributed to the Saudisation program that is now heavily targeting health care professions such as nursing. There has been a move to establish a Bachelor qualification as a prerequisite to entry into professional nursing in Saudi Arabia (Abo Zenadah, 2009).

The results indicate that, in general, Saudis are motivated to join the nursing profession for reasons similar to those of other nurses around the world and that there was very little difference between the genders in these motivations. Helping others to cope with illnesses, being in a caring occupation, and altruism were the strongest motivations for Saudi nurses to embark on a career in nursing. These results are supported by the literature. In Mebrouk's [20] study, conducted in a similar context, investigating the perceptions of nursing care among female Saudi nurses, they were motivated to pursue a career in nursing based on their desire to help people. De Cooman et al.'s [7] study investigated the motives driving younger people in Belgium to become nurses; they found that respondents intending to pursue a nursing career indicated altruism and helping people as their greatest motivators. Another study in Australia also found that individuals who chose to become nurses were motivated by working in a caring profession and altruism [21]. In the UK, altruism also was the most frequently cited reason for joining the nursing profession [10].

Job security and job flexibility were also identified in this study as motivating Saudis to join the nursing field. These findings are supported by the results of other studies in different settings [21–23]. However, other studies found that males were more motivated than females to pursue job security and flexibility (Boughn, 1994, 2001; [24, 25]). This is in contrast to the finding of this study which did not find a difference between genders. In this study, the factors which motivated Saudis the least to join the nursing profession were family expectations and advice from friends. According to Tumulty [4], nursing ranked last as an appropriate occupation for Saudi Arabians. Previously it was identified that, in Saudi society, both men and women face criticism from family and friends if they choose nursing as a career [26]. This would also explain why family expectations were found to be the least motivating factor for Saudis nurses in this study. The limited existing literature indicates that in general Saudis are not motivated to become nurses [5, 27, 28]. However, the significant increase in the number of Saudis, especially men, joining the nursing profession in recent years indicates that a change is occurring within Saudi Arabia. However, only 25% of the respondents to the survey were males; this may be because large numbers of Saudi male nurses choose to work in Primary Health Care Centres (anecdotal evidence).

### 4.1. Perceptions on Nursing

The majority of Saudi nurses who responded in this study view nursing as a caring profession, yet a stressful one that keeps them away from home for long hours. Caring is part of the culture of the Saudi community and is a central tenet of Islamic teachings [29]. The literature suggests that among nurses and, in particular, new graduates the general perception of nursing is that it is a caring profession [13, 22, 30] and that this is often a strong motive for individuals to choose nursing as a profession [21].

The respondents in this study considered nursing a stressful career; this is reflected globally in studies regarding this issue [31–33]. The relative youth and inexperience of respondents in this study may account for the high number who indicated they believed nursing is a stressful profession. Research indicates that nurses of all ages regard the profession as stressful and one which requires high levels of physical and emotional strength; however, this is felt more acutely, by younger nurses [13, 22].

Historically in Saudi Arabia, nurses' roles have been viewed as an extension of or ancillary to the physicians' role [34]. This finding concurs with the results of the current study, as a considerable proportion of the respondents agreed that nurses were subservient to doctors. Gender issues in the nursing profession have been fairly extensively investigated in the literature, although not in Saudi Arabia. In Saudi Arabia, the segregation of the genders occurs in many ways; for example, female patients prefer to receive care from female nurses and in most cases do not accept care provided by men, whether they are nurses or physicians. In addition, female patients admitted to hospitals are placed in designated female wards staffed exclusively by female nurses, although they can include physicians of both genders. Previous studies illustrate that there is a high demand for male nurses, but these studies do not address whether male nurses can be supplied in the required numbers [35, 36]. As discussed men comprise 25% of the total nursing workforce and 50% of all Saudi nurses [2]. Literature indicates in another Muslim country, Jordan, there is also a reasonably balanced gender distribution in nursing (in 2006, 40% of nurses were male) [36]. The more balanced pattern of gender distribution in nursing in Saudi Arabia, compared to many Western countries, may be due to cultural, religious, and health care demands.

### 4.2. Recruitment and Retention

Recruitment and retention of nurses are global dilemmas. Since the 1980s, internationally the nursing field has faced recruitment and retention difficulties (Ritter, 2011; Roberts, 1988). The Saudi health care system is no different. The results of this study indicate that a significantly large proportion of the respondents intend to leave the nursing profession in the near future. This is supported by the findings of another Saudi study which demonstrated that 50% of Saudis who graduate as nurses leave the profession soon after graduation, due to social and professional issues [37]. In comparison, only 9% of nurses, in a study across ten European countries, intended to leave the profession [38]. A number of factors affecting the recruitment and retention of local nurses have been noted in the literature; these factors include the negative social image associated with nursing, the nature of nursing work in Saudi hospitals, a lack of awareness about the nursing profession among young Saudis, the absence of professional development opportunities, and a lack of support for working mothers (Abu-Zinadah, 2004; Al-Sa'd, 2007). The results of this study also indicate that Saudi nurses intend to leave the profession, due to a desire to undertake further education, lack of promotion opportunities, and the requirement to work long hours. However, although gender issues are not significant to the majority of nurses, for many (more than a third) these remain important considerations and it is vital to support new nurses in adapting to a Western style health system and to where possible maintain culturally appropriate systems. The government in Saudi Arabia has recently made improvements to nursing education by increasing the number of nursing schools and places for Saudis in higher education. However, the results indicate that further strategies must be implemented that address the retention of Saudi nurses and that this is crucial to ensuring a sustainable nursing workforce for the future [39, 40]. One of the key findings was the dissatisfaction with working hours. Introduction of more flexible working conditions and part time options may help retain nurses in the workforce.

The limitations of the study are that it was only conducted in Riyadh and only within MOH hospitals. It may therefore not be generalisable to the entire Saudi nursing workforce. There may be differences particularly in regard to patterns of recruitment and retention of Saudi nurses within the public sector and between public and private sectors. The other limitation is that the survey did not access those nurses who have left the profession. Future research on this matter may give insight into this issue.

## 5. Conclusion

In conclusion the number of Saudi men entering the nursing profession in Saudi Arabia has been increasing significantly over the last decade, resulting in a balanced gender distribution across the nursing workforce. The results indicate that an increase in the recruitment of Saudi males may simply reflect cultural issues such as gender specific facilities and the Saudisation program's nondiscriminatory approach to employment of both genders into nursing. The main motivations for Saudis to enter the profession may be considered idealistic, including altruism, caring for others, and wanting to help others. The second group of motivations are more pragmatic and are centred on issues such as job security, flexibility, and career advancement. It appears that a large number of nurses are considering leaving the profession in the near future and the main factors influencing this decision are working hours, opportunities for promotion, and the desire to study. Successful recruitment without the corresponding appropriate level of attention to retention will not achieve the required nursing workforce in Saudi Arabia. For this reason it is important to explore these issues in greater depth, particularly by focusing on recent nursing graduates of Saudi nationality.

## Supplementary Material

Supplementary material 1/appendix 1 is the english version of the questionnaire.
Supplementary material 2/appendix 2 is the arabic version of the questionnaire.

## Figures and Tables

**Table 1:** Respondents' highest qualification.

	Certificate of nursing	Diploma of nursing	Bachelor nursing	Master of nursing	Ph.D.
Males	0	73.8% *n* = 138	17% *n* = 32	7.5%	1
Female	0	85% *n* = 468	13% *n* = 75	7%	13

**Table 2:** Respondents years of experience.

Experience (years of experience)	Number (%)	Percentage distribution
Less than 1 year	180	24.3%
1–5	393	53.1%
6–10	89	12%
11–15	35	4.7%
More than 15 years	43	5.8%

Total	740	100%

**Table 3:** Motivations to become a nurse.

I became a nurse because	Strongly agree	Agree	Undecided	Disagree	Strongly disagree
(1) being altruistic “Ethar” is part of Islam teachings	40.6% *n* = 301	39.1% *n* = 290	10% *n* = 74	4.6% *n* = 34	1.3% *n* = 10

(2) I wanted to work in a caring occupation	45.5% *n* = 337	46.6% *n* = 345	3% *n* = 22	3.8% *n* = 28	.5% *n* = 4

(3) I wanted to help others cope with illness	61.7% *n* = 457	32.7% *n* = 242	2.6% *n* = 19	1.5% *n* = 11	.4% *n* = 3

(4) it would give my life a sense of meaning	49% *n* = 363	36% *n* = 267	8.8% *n* = 65	4.5% *n* = 33	1.1% *n* = 8

(5) I wanted to help people	58.8% *n* = 436	37.8% *n* = 280	.8% *n* = 6	1.5% *n* = 11	.3% *n* = 2

(6) I felt that it would provide an opportunity for career advancement	46% *n* = 341	37.2% *n* = 276	9.2% *n* = 68	4.9% *n* = 36	1.6% *n* = 12

(7) nursing offered job security	36.3% *n* = 269	41% *n* = 304	12.1% *n* = 90	6.9% *n* = 51	2.4% *n* = 18

(8) I was always interested in science	42.5% *n* = 315	42.4% *n* = 314	7.3% *n* = 54	5.7% *n* = 42	.8% *n* = 6

(9) nursing offered job flexibility	31.4% *n* = 233	42.8% *n* = 317	11.2% *n* = 83	10.0% *n* = 74	3% *n* = 22

(10) I could earn a good salary	19.7% *n* = 146	43.7% *n* = 324	11.3% *n* = 84	19% *n* = 141	5% *n* = 37

(11) I like working with people	35.2% *n* = 261	51.4% *n* = 381	6.2% *n* = 46	4.3% *n* = 32	.7% *n* = 5

(12) it was a childhood desire	28.9% *n* = 214	27% *n* = 200	14.3% *n* = 106	19.3% *n* = 143	9.2% *n* = 68

(13) it was a family expectation	16.6% *n* = 123	27.0% *n* = 200	20.6% *n* = 153	23.8% *n* = 176	10.5% *n* = 78

(14) of advice from family	18.8% *n* = 13	32.3% *n* = 239	11.7% *n* = 87	24.7% *n* = 183	10.3% *n* = 76

(15) of advice from friend	13.2% *n* = 98	23.2% *n* = 172	13.8% *n* = 102	35.6% *n* = 264	12.7% *n* = 94

(16) of advice from nurse	11.3% *n* = 84	17.8% *n* = 132	13.9% *n* = 103	39.9% *n* = 296	13.9% *n* = 103

(17) of personal experience of health care	15.8% *n* = 117	28.9% *n* = 214	13.9% *n* = 103	28.6% *n* = 212	10.7% *n* = 79

**Table 4:** Perceptions of nursing as a profession.

In my opinion nursing	Strongly agree	Agree	Undecided	Disagree	Strongly disagree
(1) is caring profession	66.0% *n* = 489	29.8% *n* = 221	1.9% *n* = 14	.7% *n* = 5	.3% *n* = 2

(2) is for women	15.2% *n* = 113	21.9% *n* = 162	8.1% *n* = 60	37.8% *n* = 280	16.1% *n* = 119

(3) requires physical activity	36.6% *n* = 271	46.0% *n* = 341	5.1% *n* = 38	8.8% *n* = 65	1.8% *n* = 13

(4) is a profession that is subservient to doctors	13.8% *n* = 102	25.1% *n* = 186	7.0% *n* = 52	37.7% *n* = 279	14.8% *n* = 110

(5) does not require high academic qualifications	10.1% *n* = 75	20.1% *n* = 149	8.4% *n* = 62	33.7% *n* = 250	26% *n* = 193

(6) is a stressful career	47.1% *n* = 349	39.3% *n* = 291	3.2% *n* = 24	7.0% *n* = 52	2.3% *n* = 17

(7) offers variety	35.8% *n* = 265	49.4% *n* = 366	6.7% *n* = 50	5.3% *n* = 39	1.6% *n* = 12

(8) is a respected profession	19.6% *n* = 145	26.9% *n* = 199	15.1% *n* = 112	25.9% *n* = 192	12% *n* = 89

(9) is well paid	18.1% *n* = 134	55.3% *n* = 410	9.3% *n* = 69	10.7% *n* = 79	5.7% *n* = 42

(10) requires you to be away from home for long time	52.1% *n* = 386	36.4% *n* = 270	2.4% *n* = 18	5.5% *n* = 41	3% *n* = 22

**Table 5:** Reasons for considering leaving nursing.

The reason that I would leave is	Strongly agree	Agree	Undecided	Disagree	Strongly disagree
(1) my gender	21.3% *n* = 56	22.4% *n* = 59	9.1% *n* = 24	31.2% *n* = 82	16% *n* = 42

(2) dealing with the opposite sex	16.1% *n* = 43	31.8% *n* = 85	5.6% *n* = 15	32.2% *n* = 86	14.2% *n* = 38

(3) I feel other nurses are not comfortable with me	11% *n* = 29	11.4% *n* = 30	15.9% *n* = 42	38.3% *n* = 101	23.5% *n* = 62

(4) I feel uncomfortable dealing with nurses of the opposite sex	16.2% *n* = 43	20.3% *n* = 54	5.6% *n* = 15	40.6% *n* = 108	17.3% *n* = 46

(5) I feel uncomfortable dealing with patients of the opposite sex	16.7% *n* = 44	23.1% *n* = 61	6.8% *n* = 18	38.3% *n* = 101	15.2% *n* = 40

(6) I feel uncomfortable dealing with physicians from the opposite sex	13.7% *n* = 36	15.2% *n* = 40	8.7% *n* = 23	45.6% *n* = 120	16.7% *n* = 44

(7) I am moving away	21.7% *n* = 57	24.3% *n* = 64	17.1% *n* = 45	25.9% *n* = 68	11% *n* = 29

(8) I will become a full time student	36.6% *n* = 96	22.1% *n* = 58	19.5% *n* = 51	15.3% *n* = 40	6.5% *n* = 17

(9) Lack of promotion opportunities	34.1% *n* = 90	25% *n* = 66	14.8% *n* = 39	18.2% *n* = 48	8% *n* = 21

(10) I found a better job	22% *n* = 58	15.5% *n* = 41	19.3% *n* = 51	28.4% *n* = 75	14.8% *n* = 39

(11) I have to work long hours	65% *n* = 173	21.1% *n* = 56	3.4% *n* = 9	8.3% *n* = 22	2.3% *n* = 6

(12) I am having difficulties in communicating in English	17.6% *n* = 47	18% *n* = 48	4.9% *n* = 13	33.3% *n* = 89	26.2% *n* = 70

**Table 6:** Changes which would impact on decision to leave the profession.

Rank	Changes which would make nurses reconsider leaving the profession	Frequency of responses
(1)	Decreasing the working hours.	75
(2)	Access to continuing education and scholarships.	51
(3)	Improving the image of nursing and respect for nurses.	39
(4)	Salary increases.	38
(5)	Providing support services for nurses such as nursery and transport.	33
(6)	Decreasing workload.	24
(7)	Improving the working environment.	14
(9)	Separating genders in the work place.	13
(10)	Facilitating transfer between departments and disciplines.	9
