# Supplementary material for: The Profile of Saudi Nursing Workforce: A Cross-Sectional Study
Source: Nurs Res Pract. 2017 Oct 29;2017:1710686. doi: 10.1155/2017/1710686 (PMC5682069; doi:10.1155/2017/1710686)
Supplement: Supplementary file 1 — Supplementary material 1/appendix 1 is the english version of the questionnaire. Supplementary material 2/appendix 2 is the arabic version of the questionnaire. [file 1710686.f1.zip › Supplementary Material/appendix 1.pdf]

A descriptive study of the Saudi nursing workforce in Riyadh, Saudi Arabia

Section A

Demographic Data:

Please tick the correct answer

1. Are you a Saudi Arabian nationality:

   Yes ☐ No ☐

2. Your age in years:

   ☐☐

3. Gender:

   Male ☐ Female ☐

4. Marital status:

   Married ☐ Single ☐

5. Dependants:

   No children ☐ 1-2 children ☐ More than 2 ☐

6. Province of origin:

   Central ☐ East ☐ West ☐ North ☐ South ☐

7. Highest nursing qualification:

   Certificate ☐ Diploma ☐ Bachelor ☐ Master ☐ PhD ☐

8. Years of nursing experience:

   < 1 ☐ 1-5 ☐ 6-10 ☐ 11-15 ☐ > 15 ☐

9. Position held:
Please indicate by ticking the position in which you are currently employed. If you hold more than one position, please tick the position in which you are predominantly employed.

**Clinical**		**Education**		**Management**	
Registered Nurse	☐	Nurse Academic	☐	Nursing Unit Manager	☐
Clinical Nurse Specialist	☐	Clinical Nurse Educator	☐	Asst Director of Nursing	☐
Midwife Nurse	☐	Educator	☐	Deputy Director of Nursing	☐
Clinical Nurse Consultant	☐	Senior Nurse Educator	☐	Director of Nursing	☐
		Staff Development	☐	Area DON	☐
				Manager Nurse Education	☐
				Area Manager Nurse Ed.	☐

If 'Other' please specify ☐

10. Principal Area of Nursing Practice
Please indicate by ticking the principal area of nursing practice in which you are currently employed. If you are currently working in more than one area of practice, please tick the area in which you are predominantly employed.

Medical	☐	Surgical	☐	Nursing Education	☐
Emergency	☐	Intensive care	☐	Mental Health	☐
Midwifery	☐	Community Care	☐	Paediatric	☐
OPD	☐				

If 'Other' please specify ☐

Section B

Motivation to become a nurse:

Please respond to the statements by ticking the box that best describes your level of agreement.

I became a nurse because:		Strongly Agree	Agree	Un decided	Disagree	Strongly Disagree
1.	being altruistic " Ethar" is part of Islam teachings					
2.	I wanted to work in a caring occupation					
3.	I wanted to help others cope with illness					
4.	it would give my life a sense of meaning					
5.	I wanted to help people					
6.	I felt that it would provide an opportunity for career advancement					
7.	nursing offered job security					
8.	I was always interested in science					
9.	nursing offered job flexibility					
10.	I could earn a good salary					
11.	I like working with people					
12.	it was a childhood desire					
13.	it was a family expectation					
14.	of advice from family					
15.	of advice from friend					
16.	of advice from nurse					
17.	of personal experience of healthcare					

If other reason, please specify: ____

Section C

Perception of nursing:

Please respond to the statements by ticking the box that best describes your level of agreement.

In my opinion, nursing:	Strongly Agree	Agree	Un decided	Disagree	Strongly Disagree
1. is caring profession					
2. is for women					
3. requires physical activity					
4. is a profession that is subservient to doctors					
5. does not require high academic qualifications					
6. is a stressful career					
7. offers variety					
8. is a respected profession					
9. is well paid					
10. requires you to be away from home for long time					

Section D

Future plans:

1. As a Saudi nurse, would you prefer to work part time

   Yes ☐ No ☐

2. If yes, how many days a week would you prefer to work?

   ☐ Days

3. Would you prefer to work shorter shifts?

   Yes ☐ No ☐

4. Do you intend to leave nursing in the near future (< 2 years)?

   Yes ☐ No ☐

   If **yes**, please go to Question (6), if **No**, please go to question (5).

5. If you are not intending to leave nursing in 2 years, would you like to leave nursing?

   Yes ☐ No ☐

   If **yes**, please go to Question (6), if **No**, this is the **END** of the survey for you thanks for your time.

6. If it is likely that you will leave nursing, please indicate your level of agreement for the following statements:

**The reason that I would leave is:**		**Strongly Agree**	**Agree**	**Un decided**	**Disagree**	**Strongly Disagree**
1.	my gender.					
2.	dealing with the opposite sex.					
3.	I feel other nurses are not comfortable with me.					
4.	I feel uncomfortable dealing with the opposite sex.					
5.	I feel uncomfortable dealing with nurses from the opposite sex.					
6.	I feel uncomfortable dealing with patient from the opposite sex.					
7.	I feel uncomfortable dealing with physicians from the opposite sex.					
8.	I am moving away.					
9.	I will become a full time student.					
10.	lack of promotion opportunities.					
11.	I found a better job.					
12.	I have to work long hours.					
13.	I am having difficulties in communicating in English.					

If other reason, please specify: ____

7. Is there anything that could be changed which would influence your decision to leave nursing? Please write you answer below

End of the Survey

Thank you for participating in this study and for your time.

Mohammad Alboliteeh
