# Supplementary material for: The Profile of Saudi Nursing Workforce: A Cross-Sectional Study
Source: Nurs Res Pract. 2017 Oct 29;2017:1710686. doi: 10.1155/2017/1710686 (PMC5682069; doi:10.1155/2017/1710686)
Supplement: Supplementary file 1 — Supplementary material 1/appendix 1 is the english version of the questionnaire. Supplementary material 2/appendix 2 is the arabic version of the questionnaire. [file 1710686.f1.zip › Supplementary Material/appendix 2.pdf]

دراسة وصفية للقوى العاملة السعودية في مجال التمريض بمدينة الرياض، المملكة العربية السعودية

القسم الأول: البيانات الديمغرافية

الرجاء وضع علامة ✓ أمام الإجابة التي ترونها صحيحة

١. هل أنت سعودي الجنسية:

نعم ☐ لا ☐

٢. العمر بالسنوات:

☐☐

٣. الجنس:

ذكر ☐ أنثى ☐

٤. الحالة الاجتماعية:

متزوج ☐ غير متزوج ☐

٥. عدد الأطفال:

بدون ☐ ١-٢ طفل ☐ أكثر من طفلين ☐

٦. المنطقة التي تنتمي لها (نشأت بها):

الوسطى ☐ الشرقية ☐ الغربية ☐ الشمالية ☐ الجنوبية ☐

٧. أعلى مؤهل دراسي حصلت عليه في مجال التمريض:

دورة ☐ دبلوم ☐ بكالوريوس ☐ ماجستير ☐ دكتوراه ☐

٨. عدد سنوات الخبرة في مجال التمريض:

اقل من سنه ☐ ١-٥ سنوات ☐ ٦-١٠ سنوات ☐ ١١-١٥ سنة ☐ أكثر من ١٥ سنة ☐

THE UNIVERSITY of ADELAIDE

٩. المنصب الوظيفي الحالي:

الرجاء وضع علامة ✓ أمام المنصب الذي تشغلونه حاليا. وفي حالة أنكم تشغلون أكثر من منصب فأرجو الإشارة إلى منصبكم حسب ملفكم الوظيفي.

**المجال ألسريري**	**المجال التعليمي**	**المجال الإداري**
ممرض / ممرضة مسجل ☐	ممرض أكاديمي ☐	مدير وحدة تمريض ☐
ممرض/ ممرضة أخصائي ☐	مدرب تمريض سريري ☐	مساعد رئيس تمريض ☐
ممرضة قابلة ☐	مدرب تمريض ☐	نائب رئيس تمريض ☐
ممرض/ ممرضة استشاري ☐	رئيس مدربي تمريض ☐	رئيس التمريض ☐
	مسئول تطوير المهارات ☐	رئيس منطقة تمريض ☐
		مدير تدريب التمريض ☐

إذا أخرى فأرجو التوضيح ☐

١٠. المجال الرئيسي لممارسة التمريض:

الرجاء وضع علامة ✓ أمام الوحدة التي تعملون فيها حاليا. وفي حالة أنكم تعملون في أكثر من مجال أو قسم في وقت واحد فأرجو الإشارة إلى مجالكم الرئيسي حسب ملفكم الوظيفي.

الرعاية الباطنية ☐	الرعاية الجراحية ☐	تعليم التمريض ☐
الطوارئ ☐	العناية المركزة ☐	الصحة النفسية ☐
النساء والولادة ☐	صحة المجتمع ☐	الأطفال وحديثي الولادة ☐
العيادات الخارجية ☐		

إذا أخرى فأرجو التوضيح ☐

THE UNIVERSITY of ADELAIDE

SUB CRUCE LUMEN

القسم الثاني: الدوافع والمحفزات للالتحاق بمجال التمريض

الرجاء الإجابة على الجمل التالية بوضع علامة ✓ أمامها بما يتوافق مع مستوى موافقتكم عليها.

**لقد انخرطت في مجال التمريض بسبب:**	**أوافق بشدة**	**أوافق**	**لا أعلم**	**لا أوافق**	**لا أوافق بشدة**
١. أنني أعتبر أن إيثار الغير على النفس من أهم تعاليم الإسلام					
٢. رغبتي في العمل في مهنة تهتم بالرعاية					
٣. رغبتي في مساعدة الآخرين على التغلب على المرض					
٤. أنني رأيت أن هذه المهنة سوف تعطي لحياتي معنى آخر					
٥. رغبتي في مساعدة الآخرين					
٦. شعوري بأنه سوف يمنحني فرصة للتقدم الوظيفي في المستقبل					
٧. أن مهنة التمريض تمنح أمان وظيفي					
٨. أنني كنت دائما أرغب في العمل بالمجال العلمي					
٩. أن مهنة التمريض تقدم مرونة أكثر في العمل					
١٠. أنها مهنة تحقق لي راتب مغري					
١١. أنني أرغب في العمل مع الآخرين					
١٢. أنها كانت طموحي منذ صغري					
١٣. أنها طموح أسرتي					
١٤. أنها نصيحة من أسرتي					
١٥. أنها نصيحة من صديق					
١٦. أنها نصيحة من ممرض					
١٧. خبرة وتجربة شخصية في مجال العمل بالرعاية الصحية					

لسبب آخر(وضح) ______________________

القسم الثالث: التصور والنظرة لمهنة التمريض

الرجاء الإجابة على الجمل التالية بوضع علامة ✓ أمامها بما يتوافق مع مستوى موافقتكم عليها.

من وجهة نظري فإن مهنة التمريض:	أوافق بشدة	أوافق	لا أعلم	لا أوافق	لا أوافق بشدة
١. مهنة تعتمد على الرعاية والعناية					
٢. مهنة تخص النساء					
٣. مهنة تحتاج إلى قدرات بدنية					
٤. مهنة خاضعة لتنفيذ تعليمات وأوامر الأطباء فقط					
٥. مهنة لا تحتاج إلى مؤهلات أكاديمية عالية					
٦. مهنة فيها الكثير من الإرهاق					
٧. مهنة تمنح الكثير من التنوع العملي					
٨. مهنة يحترمها الجميع					
٩. مهنة راتبها جيد					
١٠. تتطلب الابتعاد عن المنزل والعائلة لفترات طويلة					

القسم الرابع: الخطط المستقبلية

١. هل تفضل/ تفضلين العمل بطريقة الدوام الجزئي؟ (أيام متفرقة في الأسبوع وليس الأسبوع كاملا)

نعم ☐ لا ☐

٢. إذا كان الجواب نعم فكم يوما تفضل/ تفضلين العمل في الأسبوع؟

☐ يوم

٣. هل تفضل/ تفضلين العمل ساعات أقل مما هي عليه حاليا؟

نعم ☐ لا ☐

٤. هل لديك النية (تخطط) لترك العمل في مجال التمريض في المستقبل القريب (اقل من سنتين)؟

نعم ☐ لا ☐

إذا كان الجواب **نعم** فأذهب إلى سؤال رقم(٦)، وإذا كان الجواب **لا** فأذهب إلى سؤال رقم (٥)

٥. إذا لم تكن لديك النية لترك العمل في مجال التمريض ، فهل لديك الرغبة في (تتمنى) تركه؟

نعم ☐ لا ☐

إذا كان الجواب **نعم** فأذهب إلى سؤال رقم(٦)، وإذا كان الجواب **لا** فهذه هي نهاية الاستبيان بالنسبة لك شكرا لك على وقتك.

٦. إذا كانت لديك النية/ الرغبة لترك التمريض فأرجو الإجابة على الجمل التالية بوضع علامة ✓ أمامها بما يتوافق مع مستوى موافقتكم عليها.

**السبب في نيتي/ رغبتي ترك مهنة التمريض هو:**	**أوافق بشدة**	**أوافق**	**لا أعلم**	**لا أوافق**	**لا أوافق بشدة**
١. نوعية جنسي (كوني ذكر أو أنثى)					
٢. التعامل مع أشخاص من جنس مختلف					
٣. إحساسي بأن الآخرين لا يشعرون بالارتياح لي					
٤. أنني لا أحس بالارتياح في التعامل مع الجنس الأخر					
٥. عدم ارتياحي في التعامل مع **ممرضين** من الجنس الأخر					
٦. عدم ارتياحي في التعامل مع **مرضى** من الجنس الأخر					
٧. عدم ارتياحي في التعامل مع **أطباء** من الجنس الأخر					
٨. أنني سأنتقل لمكان أخر					
٩. أنني سأتفرغ للدراسة					
١٠. قلة فرص الترقية					
١١. لاني وجدت وظيفة أفضل من التمريض					

تابع للسؤال رقم (٦)

**السبب في نيتي/ رغبتي لترك مهنة التمريض هو:**	**أوافق بشدة**	**أوافق**	**لا أعلم**	**لا أوافق**	**لا أوافق بشدة**
١٢. طول ساعات العمل في مجال التمريض					
١٣. أنني أواجه صعوبة في التعامل باللغة الإنجليزية					

لسبب أخر (وضح) : ________________________

٧. هل هناك شي يمكن فعله من شأنه أن يجعلك تغير قرارك لترك مهنة التمريض؟ نرجو كتابة إجابتك.

____

____

____

____

____

نهاية الاستبيان

أشكر لك وقتك وحسن استجابتك للمشاركة في ملئ الاستبيان.

محمد إبراهيم البليطيح
جامعة أديليد- أستراليا
